# Origin of high oxygen reduction reaction activity of Pt_12_ and strategy to obtain better catalyst using sub-nanosized Pt-alloy clusters

**DOI:** 10.1038/srep45381

**Published:** 2017-03-28

**Authors:** Kasumi Miyazaki, Hirotoshi Mori

**Affiliations:** 1 Department of Chemistry and Biochemistry, Graduate School of Humanities and Sciences, Ochanomizu University, 2-1-1 Otsuka, Bunkyo-ku, Tokyo 112-8610, Japan; 2 Faculty of Core Research Natural Science Division, Ochanomizu University, 2-1-1 Otsuka, Bunkyo-ku, Tokyo 112-8610, Japan

## Abstract

In the present study, methods to enhance the oxygen reduction reaction (ORR) activity of sub-nanosized Pt clusters were investigated in a theoretical manner. Using ab initio molecular dynamics and Monte Carlo simulations based on density functional theory, we have succeeded in determining the origin of the superior ORR activity of Pt_12_ compared to that of Pt_13_. That is, it was clarified that the electronic structure of Pt_12_ fluctuates to a greater extent compared to that of Pt_13_, which leads to stronger resistance against catalyst poisoning by O/OH. Based on this conclusion, a set of sub-nanosized Pt-alloy clusters was also explored to find catalysts with better ORR activities and lower financial costs. It was suggested that Ga_4_Pt_8_, Ge_4_Pt_8_, and Sn_4_Pt_8_ would be good candidates for ORR catalysts.

The oxygen reduction reaction (ORR) is one of the most important chemical reactions for realizing clean energy technology. One of the main problems for the application of the ORR is the slowness of the reaction^1^. In order to accelerate the reaction, Pt-based materials have generally been used as catalysts^2^. However, they are expensive, not efficient enough for practical use, or both. Many researchers have engaged in finding materials with higher catalytic performances and lower financial costs. In the case of heterogeneous catalysts, e.g., Pt-based materials, the surface area has been considered a key parameter in controlling the activities. Since smaller particles have larger surface areas with respect to their volumes, their mass catalytic activities should be higher than those of larger ones. Thus, nano-sizing of Pt particles has been considered as one of the methods to increase their surface areas. However, the reality is not so simple. Indeed, the ORR catalytic activities of Pt nanoparticles are known to increase as their sizes decrease, but the activity decreases when the particle size is smaller than 2 nm^3^,^4^,^5^. This is because a smaller particle has a larger number of edges and vertices that interact very strongly with O/OH, leading to surface poisoning^3^. If we want to increase ORR catalytic activities with nano-sized Pt particles, we have to consider both the surface area and the poisoning effect.

Based on this background knowledge, the minimum Pt nanoparticle radius for ORR catalysts had been considered to be ca. 2 nm until Yamamoto, Imaoka *et al*. found that sub-nanosized Pt clusters showed extremely high ORR activities.^6^,^7^,^8^,^9^. The authors reported that sub-nanosized Pt clusters (Pt_*n*_; *n* = 12–20), the atomicities of which were precisely defined, showed higher activity than bulk Pt and 2 nm nanoparticles, larger-size Pt particles containing thousands of atoms. Furthermore, they revealed that the catalytic activity of Pt_12_ is two times higher than that of Pt_13_, although the difference in the number of atoms is only one^6^. Generally, the activities of catalytic materials depend on their electronic structures. These experimental results implied that there should be differences in the electronic structures of Pt_12_ and Pt_13_. In order to clarify these differences, information on the three-dimensional geometries of the materials is essential, since their electronic structures are governed by their cluster geometries. However, the geometries of sub-nanosized Pt clusters have not been observed directly. Scanning transmission electron microscopy, for example, can measure only two-dimensional geometries of sub-nanosized clusters^7^. Therefore, their electronic structures and the relationship between their structures and catalytic activities are unclear.

As experimental methods such as X-ray diffraction cannot be applied to investigate sub-nanosized Pt clusters, we have to investigate them using theoretical methods. In order to obtain a plausible theoretical geometry for each metallic cluster, however, it is necessary to use global optimization, which is a method that determines the most stable geometry in a multi-dimensional potential energy surface for a given system^10^,^11^,^12^,^13^. For an *N*-atom system, the dimension of the structural phase space is 3 *N*. The number of local minima is known to increase exponentially with respect to *N*^14^,^15^. Generally speaking, it is impossible to find all stable potential minima in 3*N*-dimensional space using a simple geometry optimization technique^16^. Furthermore, local optimizations from nonsensical initial geometries are often trapped in local minima with high energy; however, this is not important for discussions on catalysis. Moreover, the experimentally observable physicochemical properties of each cluster cannot be entirely understood through only a global minimum geometry owing to the existence of minima with similar potential energies^17^,^18^. For example, Rodríguez-Kessler *et al*. have mentioned that O/OH adsorption energies for Pt_12_/Pt_13_ depend on the cluster geometries, i.e., fluctuation^19^. It should be noted that the adsorption energies have often been utilized as simple indicators of ORR activity^20^,^21^,^22^. Previous reports have clearly shown that geometrical flexibility and the accompanying electronic fluctuation should be considered when discussing ORR activities, at least in the case of sub-nanosized Pt clusters.

The aim of this study was to identify novel sub-nanosized clusters that have higher ORR catalytic efficiencies with lower financial costs from a theoretical point of view. Among the sub-nanosized Pt clusters, we focused on Pt_12_, as the atomicity can be controlled precisely using the method developed by Yamamoto, Imaoka *et al*.^6^,^7^,^8^,^9^. Since it is not completely understood why Pt_12_ showed better ORR activity than Pt_13_ did, we focused on Pt_13_ as well. Thus, we investigated the difference between the electronic structures of Pt_12_ and Pt_13_ to reveal the cause of the difference in their ORR activities, taking the flexibility of their geometries into consideration. First, we performed ab initio molecular dynamics and simulated annealing^13^ (AIMD-SA) calculations to find a global minimum and relatively low-energy geometries for Pt_12_ and Pt_13_. The fluctuation behaviors of electronic structures within a set of stable geometries were compared. Second, to understand the difference between the electronic structures in terms of interactions with O/OH molecules that affect the rate-limiting steps of the ORR, we calculated adsorption energies for the fluctuating geometries using ab initio Monte Carlo (AIMC) simulations. Finally, based on a discussion for pure Pt clusters, we searched for several sub-nanosized alloy clusters containing Pt that can be novel catalysts with high activities and low financial costs.

## Results and Discussion

**Figure 1:**
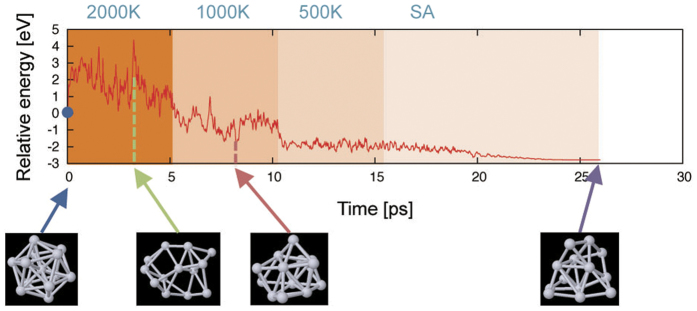
An example of AIMD-SA for Pt_13_. The vertical and horizontal axes are the energy relative to that for the initial geometry and simulation time (steps), respectively. The simulation temperature in each step is given above. The geometries depicted below represent snapshots in the AIMD-SA.

An example of AIMD-SA, in which the initial geometry belongs to the highly symmetric *I*_*h*_ point group, is shown in Fig. 1. It can be observed that the atomic configuration was well mixed to give an apparently different geometry from the initial one. The AIMD procedure succeeded in preventing the system from being trapped in local minima. It should be mentioned that the final geometry in Fig. 1 is ca. 3 eV more stable than the initial one in the *I*_*h*_ point group, which has been considered a topological *magic number*. This result indicates well the importance of finding global minima in transition metal clusters whose electronic structures are governed by complex interactions between d-orbitals.

**Figure 2:**
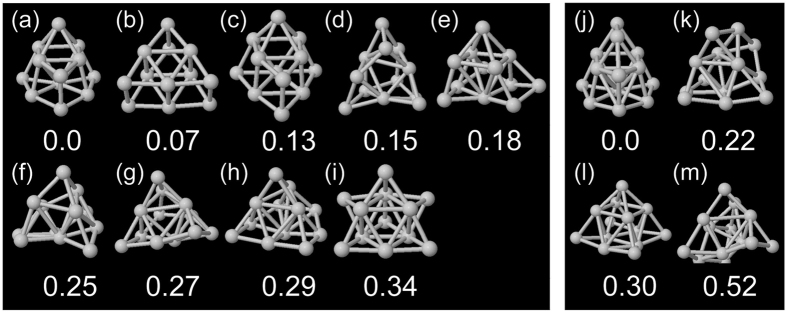
Relatively stable geometries of ((**a**–**i**)) Pt_12_ and ((**j**–**m**)) Pt_13_. The values are the energies relative to that of the most stable one (in eV).

Figure 2 shows final geometries of Pt_12_ and Pt_13_ obtained using AIMD-SA simulations started from nine different initial ones. The Cartesian coordinates of each geometry are written in the Supporting Information (SI). The energetically most probable geometries for Pt_12_ and Pt_13_ are (a) and (j), respectively. These geometries are consistent with previous global optimizations based on density functional theory (DFT) by Zhang^23^, Da Silva^24^, Wei^25^, and Zhai^26^ *et al*., which focused on obtaining a static global minimum geometry in each cluster size. Although it is difficult to obtain a global minimum with a single simulated annealing (SA) run, we can increase the precision when obtaining a global minimum geometry by performing multiple independent AIMD-SA runs. In the present study, six AIMD-SA runs with different initial conditions gave the same geometry (j) for Pt_13_, which means that it should be the global minimum^16^. It was found that both Pt_12_ and Pt_13_ have isomers whose energies are within 0.5 eV of those of the most stable ones. This indicates that the geometries of Pt_12_ and Pt_13_ fluctuate under ambient conditions. Our results clearly show the importance of the geometric fluctuations of the clusters. This agrees well with the previous report by Rodríguez-Kessler *et al*.^19^.

**Figure 3:**
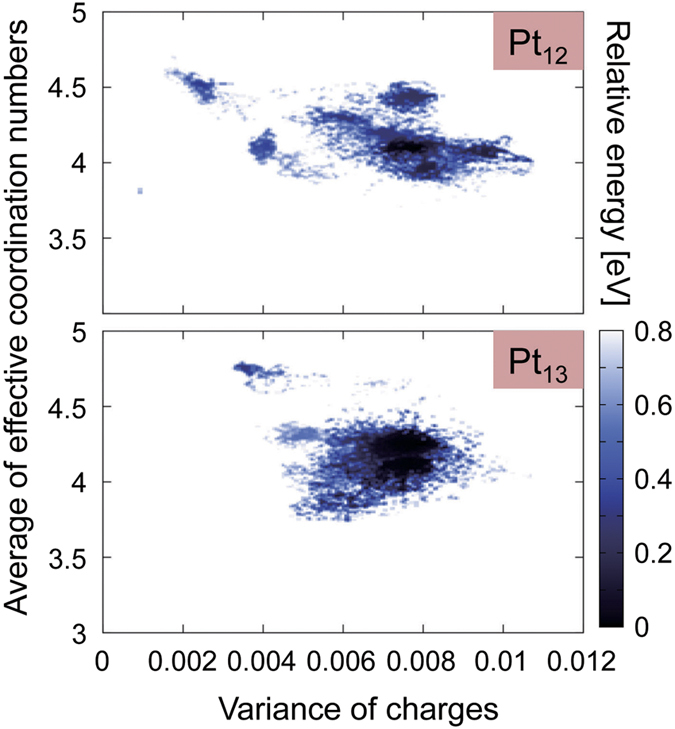
Relationship between charge variance and effective coordination number (ECN) obtained using AIMD-SA simulations. Each point corresponds to snapshot data from AIMD-SA and the shade of the plots indicates the energy relative to that of the global minimum. ECN was defined by Eqs. (1) and (2) given in the Methods section. The average ECNs (vertical) and variance of charges (horizontal) were evaluated using Eqs. (4) and (5) in the same section.

To investigate the differences in the electronic properties of Pt_12_ and Pt_13_, first, we compared their atomic natural charges^27^,^28^,^29^ and densities of states (DOSs) in the “static” global minimum geometries ((a) and (j)). Contrary to our expectations, as shown in Figures S1 and S2, we could find no apparent differences in their charge distributions. This is because we forgot that these clusters should have enough flexibility to allow their geometries to fluctuate under the experimental conditions. In order to discuss the differences between the electronic structures of Pt_12_ and Pt_13_ more precisely, it is mandatory to consider their atomic fluctuations. Next, we compared the thermal fluctuations of their electronic structures using wave functions at all atomic configurations obtained in our AIMD-SA simulations. Using this scheme, we can acquire thermally important geometries and accompanying electronic structures. Figure 3 depicts the relationship between atomic charge variance and effective coordination number (ECN)^30^ at energies less than 0.8 eV. The vertical and horizontal axes in Fig. 3 indicate the atomic fluctuation and electronic polarization in a cluster, respectively. Upon comparing the data for Pt_12_ and Pt_13_, we could observe no apparent difference in graph width in the vertical direction. This means that geometric softness, which can be viewed as atomic fluctuation, is similar for Pt_12_ and Pt_13_. On the other hand, in the horizontal direction, we could observe a clearly wider distribution for Pt_12_. Thus, we could conclude that Pt_12_ has a more flexible electronic structure than Pt_13_ does even though their degrees of atomic fluctuation are similar.

Next, we shall move our focus onto O/OH adsorption interactions on the sub-nanosized Pt clusters. To date, interaction energies of O/OH and Pt have often been used to explain their catalytic activities^20^,^21^,^22^. The relationship between the interaction energies and corresponding ORR activities has been known to give a volcano plot. This is because O and OH are key intermediate species in rate-limiting steps of the ORR. The plot tells us that the adsorption energy should not be too strong or too weak in the ORR catalytic process. Basically, the idea behind volcano plots can be well applied to describe Pt-based ORR catalytic materials. Since the basic idea of the volcano plot is based on “static” interaction between adsorbent and adsorbed species, however, strictly speaking, it should be noted that discussions involving the plot should be applied only to well-fixed solid-state materials. As explained above, the sub-nanosized Pt clusters are geometrically flexible at room temperature. Thus, there should be an enormous number of possible rate-limiting complexes with different interaction energies for the sub-nanosized Pt catalysts. In order to explain the difference between the ORR activities of Pt_12_ and Pt_13_, again, we have to consider the thermal fluctuation effect of the clusters on the adsorption interaction. In this study, we applied a set of AIMC simulations to deal with the energetic fluctuation in the adsorption interaction. The details of the AIMC simulations are described in the Methods section and SI. Using the AIMC scheme, 10^5^ geometries for each complex were sampled and the effects of thermal fluctuation were taken into account.

**Figure 4:**
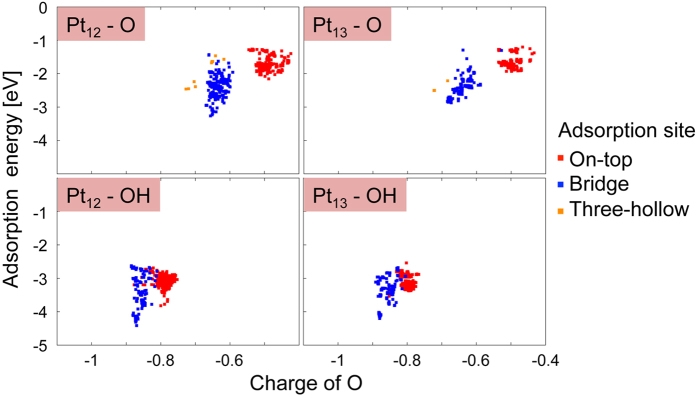
Correlation map of adsorption energy with charge on an oxygen atom in Pt_n_-O/OH (n = 12, 13).

In Fig. 4, it is shown that the O/OH adsorption energies to Pt_12_/Pt_13_ have wide energy spreads extending over 1.5 eV. In the Figure, data for different adsorption sites (i.e., on-top, bridge, and three-hollow sites) were plotted with different colors. Recently, using the global minimum geometry of Pt_13_, Chaves *et al*. showed that the adsorption energies of OH in on-top and bridge sites are −3.23 and −3.60 eV, respectively^31^, which are similar to our data in Fig. 4. We could observe that there should be a large fluctuation for the interaction energy by thermal atomic motion even in the same kind of adsorption sites. Using a few optimized geometries, Rodríguez-Kessler *et al*. showed that the adsorption energies of O/OH and Pt_12_/Pt_13_ depend on the cluster geometries^19^. Since the atomic charge of an O atom represents the amount of charge transfer between it and Pt-based ORR catalysts, it has often been considered that the atomic charge on an O atom should be a good indicator for the adsorption strength. However, in Fig. 4, where the flexibilities of the clusters are fully taken into account, we could not find such a strong correlation between the vertical and horizontal axes. Moreover, we could find no apparent difference between the degrees of atomic fluctuation (horizontal direction in Fig. 3) of Pt_12_ and Pt_13_. These results mean that a factor other than atomic fluctuation, i.e., electronic flexibility, of the sub-nanosized cluster governs the difference between the catalytic activities of Pt_12_ and Pt_13_. Table 1 shows the average and standard deviations of the O/OH adsorption energies. The standard deviations are 0.17, 0.27, 0.05, and 0.03 eV for Pt_12_-O, Pt_12_-OH, Pt_13_-O, and Pt_13_-OH, respectively. These results correspond well with the fact that Pt_12_ has a more flexible electronic structure than Pt_13_ even though they show similar magnitudes of atomic fluctuation (see Fig. 3). To put it briefly, Pt_12_ undergoes greater electronic fluctuation in O/OH adsorption than Pt_13_ does, which results in the prevention of catalyst poisoning. Our results explain well the experimental data reported by Imaoka *et al*., which indicated that Pt_12_ shows higher ORR activity than Pt_13_ does^6^.

One of the roles of current theoretical chemists is to predict novel functional materials. In this study, we also attempted to search for sub-nanosized alloy materials as candidates for ORR catalysts. As discussed above, it has been found that electronic fluctuation of sub-nanosized clusters should play an important role in preventing catalyst poisoning. Although it has been clarified that better sub-nanosized catalysts for the ORR should have large electronic fluctuations to prevent poisoning effects, random atomic fluctuation is an inconvenience in the further design of catalysts based on sub-nanosized materials. Thus, a strategy for reducing uncontrollable atomic fluctuations must be used. The geometrical flexibility of the Pt clusters originates from complex hybridizations of d-orbitals. In this paper, we propose a novel approach for developing better ORR catalysts based on the sub-nanosized Pt clusters. This approach is the sub-nanosized alloying of the Pt clusters with p-block metals. Since the valence electronic structures of p-block metals are much simpler than those of the d-block ones, the geometries of d/p-alloyed clusters are expected to have moderately fixed atomic configurations and large electronic fluctuations. In this study, we explored the possibility of using M_4_Pt_8_ (M = Al, Ga, Ge, and Sn) as ORR catalysts using the AIMD-SA and AIMC scheme mentioned above. Since Al, Ga, Ge, and Sn have lower electronegativities than that of Pt (1.61, 1.81, 2.06, 1.96, and 2.28, respectively), alloying them with Pt clusters is expected to enhance electronic polarization and fluctuation in the electronic structure of the alloy clusters. At the same time, as these metals are much cheaper than Pt is, these M_4_Pt_8_ (M = Al, Ga, Ge, and Sn) alloy clusters are good candidates for ORR catalysts from an elemental strategy point of view. The number of heteroatoms here was chosen to suit the experimental synthesis technique developed by Yamamoto, Imaoka *et al*.^6^,^7^,^8^,^9^.

**Figure 5:**
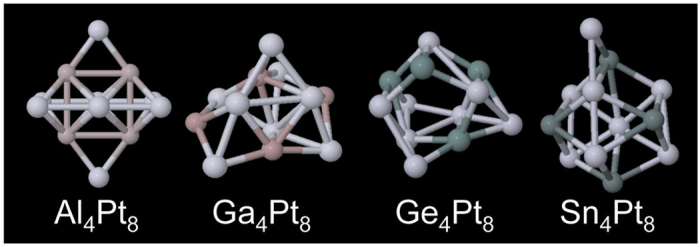
Most stable geometry of each cluster.

**Figure 6:**
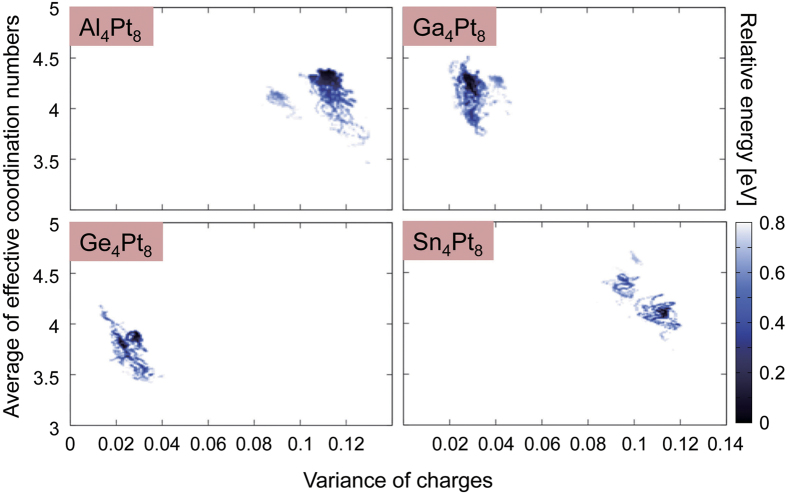
Relationships between charge variance and ECN obtained using AIMD-SA simulations. Each point corresponds to snapshot data from AIMD-SA and the shade of the plots indicates the energy relative to that of the global minimum. The ECN was defined by Eqs. (1), (2), 3 given in the Methods section. The average ECNs (vertical) and variance of charges (horizontal) were evaluated using Eqs. (4) and (5) in the same section.

The most stable geometries obtained for M_4_Pt_8_ (M = Al, Ga, Ge, and Sn) are depicted in Fig. 5. For Al_4_Pt_8_, we could obtain only one geometry even though we performed several AIMD-SA runs with different initial conditions. This indicates that this geometry is most likely to be a global minimum^16^. For Ga_4_Pt_8_, Ge_4_Pt_8_, and Sn_4_Pt_8_, we found a few local minima within the energy range of 0.3 eV (data not shown). Figure 6 shows the relationships between geometric and electronic fluctuations for M_4_Pt_8_ (M = Al, Ga, Ge, and Sn). It should be noted that the vertical axis of Fig. 6 is ten times larger than that of Fig. 3. The variances of atomic charges are 0.11, 0.03, 0.03, and 0.11 for Al_4_Pt_8_, Ga_4_Pt_8_, Ge_4_Pt_8_, and Sn_4_Pt_8_, respectively, which are much larger than those for Pt_12_ (0.009) and Pt_13_ (0.0075). This means that polarizations of the sub-nanosized alloy clusters are larger than those of pure Pt clusters. The averaged atomic charges of the p-block metal and Pt, respectively, are + 0.46 and −0.23 for Al_4_Pt_8_, + 0.21 and −0.11 for Ga_4_Pt_8_, + 0.22 and −0.11 for Ge_4_Pt_8_, and + 0.47 and −0.23 for Sn_4_Pt_8_. The negative values of the Pt atoms in the alloy clusters can be explained reasonably using Pauling’s electronegativity. Alloying with p-block metals also enhanced charge polarization. That is, in the alloy system, electronic fluctuation is enhanced even though geometrical fluctuation is reduced.

Since we were able to control the atomic fluctuation, as expected, by introducing p-block metals, the next step was to examine the catalytic activities of the sub-nanosized alloy clusters. Figure S3 shows the adsorption energies between M_4_Pt_8_ (M = Al, Ga, Ge, and Sn) and adsorbed species (O/OH). Compared to those in Fig. 4, the distributions in Figure S3 are wider. The energy-weighted average and standard deviation of each adsorption are listed in Table 1. From these data, first, we can find that all of the alloy clusters gave similar adsorption features to those of Pt_12_/Pt_13_. This means that the alloy clusters should be candidates for ORR catalysts. However, it seems that Al_4_Pt_8_ binds with O/OH too strongly to catalyze the ORR. In the panel for Al_4_Pt_8_-OH in Figure S3, we can find adsorption sites whose adsorptions are too strong, with energies less than −4 eV (even though the energetic fluctuation is as large as that of Pt_12_). Poisoning by OH is expected to occur in Al_4_Pt_8_. For the other alloy clusters, no such poisoned site was found. Thus, it can be concluded that Ga_4_Pt_8_, Ge_4_Pt_8_, and Sn_4_Pt_8_ should be good candidates for ORR catalysts. Ge_4_Pt_8_ is especially promising because it has similar adsorption energy to Pt_12_ with larger fluctuations.

## Conclusions

The origin of the superior ORR activity of Pt_12_ to that of Pt_13_ can be explained by differences in electronic fluctuations. As Pt_12_ and Pt_13_ have numerous stable isomers, it was clarified that their physicochemical properties cannot be discussed without consideration of their atomic fluctuations. Taking their atomic fluctuations into account, we found that Pt_12_ has a more flexible electronic structure than Pt_13_ does, even though their degrees of atomic fluctuation are similar. Greater electronic fluctuation results in greater energetic fluctuation of O/OH adsorption for Pt_12_, i.e., stronger resistance against catalyst poisoning compared to Pt_13_. It was concluded that controlling the atomic/electronic fluctuations is key in realizing higher ORR catalytic activities for sub-nanosized clusters containing Pt. Based on conclusions for pure Pt clusters, we attempted to predict whether sub-nanosized M_4_Pt_8_ (M = Al, Ga, Ge, and Sn) alloy clusters, in which p-block metals were alloyed with Pt to enhance electronic flexibility and provide well-defined atomic configurations, would be good candidates for ORR catalysts. It was suggested that Ga_4_Pt_8_, Ge_4_Pt_8_, and Sn_4_Pt_8_ would show better ORR catalytic activities than Pt_12_ and Pt_13_ do and with lower costs.

## Methods

In the present study, all calculations were performed based on DFT using the TURBOMOLE 7.0 quantum chemical program package^32^. We employed the Perdew-Burke-Ernzerhof (PBE) functional^33^, which is a pure generalized gradient approximation (GGA) exchange-correlation functional, to describe the electronic structures of the target clusters. As Pt is a heavy element belonging to the fifth row in the periodic table, it is mandatory to take relativistic effects into account for sufficiently accurate quantum chemical discussions. Thus, we applied the def-SV(P) basis set with relativistic pseudopotentials^34^,^35^. Xiao *et al*. have reported that the relative energy of each isomer is not affected by spin-orbit coupling in Pt clusters^36^. Therefore, we did not include the spin-orbit coupling effect in our calculations. To reduce computational costs for two-electron integrals and to accelerate all calculations, the resolution of identity (RI) approximation was applied^37^. As our targets are sub-nanosized transition metal clusters, we had to consider their band-like (metallic) electronic structures with thermal excitation^38^. In the present study, the metallic character was described using the pseudo-Fermi smearing technique at 300 K, which is consistent with experimental conditions^39^.

To find the global and low-energy minima of Pt_12_, Pt_13_, and M_4_Pt_8_ (M = Al, Ga, Ge, and Sn), simulated annealing (SA) using ab initio molecular dynamics simulations (AIMD) was performed, in which electronic potential energy and gradient were evaluated using the quantum chemical method mentioned above. Under the experimental conditions, the sub-nanosized clusters were supported on glassy carbon^6^,^7^,^8^,^9^. According to the report by Lim *et al*., the geometries of Pt_13_ in the gas phase and on defective graphene supports are similar^40^. They also reported that ORR pathways are the same in both cases. Thus, in the present study, we used cluster models in gas-phase conditions. In the AIMD-SA simulations, the Leapfrog algorithm was applied. The details of the AIMD-SA simulations were as follows. First, a set of initial geometries was prepared. For Pt_12_ and Pt_13_, nine geometries were chosen from the previous reports by Häkkinen, in which geometries of Au_n_ (n = 4–14) were investigated using photoelectron spectroscopy and DFT calculations^41^. For the alloy clusters, M_4_Pt_8_ (M = Al, Ga, Ge, and Sn), three sets of initial geometries were defined by randomly replacing four Pt atoms in stable structures of Pt_12_ with M. Then, after generating the initial geometries, a set of the AIMD-SA simulations was performed with a time step of 10 fs for Pt_12_, Pt_13_, and M_4_Pt_8_ (M = Ga, Ge, and Sn) and 5 fs for Al_4_Pt_8_. These time steps were chosen based on one-tenth of the vibration period of Pt_2_ or PtM (M = Al, Ga, Ge, and Sn) dimers (Table 2). The AIMD-SA simulations consisted of four stages to avoid being trapped in a local minimum. In the first stage of our simulations, a set of AIMD at 2000 K was performed for 5.2 ps. The object of this stage was to scramble cluster configurations. In the second and third stages, the temperature was lowered gradually to 1000 and 500 K, respectively. In these three stages, an NVT ensemble with Nosé-Hoover thermostats was applied^42^. Finally, in the fourth stage, a set of SA runs was performed to predict a global minimum structure for each cluster. The final geometries are depicted in Figs 2 and 5.

The ECNs in Figs 3 and 6, and S4 were calculated using Eqs. (1) and (2). The ECN is a fractional number defined as the sum of weights, representing the contribution to the coordination number of the target atom^30^. The contribution of atom *j* to atom *i* is calculated based on the distance between them (*d*_*ij*_ in Eqs. (1) and (2)). The 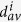 term in Eq. (2) is the average bond lengths of atom *i*. As both the left and right hand sides of Eq. (2) contain 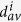
terms, Eq. (2) is solved in a self-consistent manner.


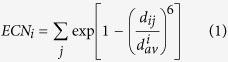



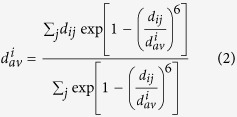


The original definition of the ECN, Eqs (1) and (2), is not suitable for alloy systems, which contain multiple types of atoms. In the present study, we replaced *d*_*ij*_ in Eqs (1) and (2) with Eq. (3) to represent the ECN of alloy systems in a suitable manner. In Eq. (3), *r*_*i*_ and *r*_*j*_ are the atomic radii of atoms *i* and *j*, respectively^43^. Upon substitution of Eq. (3) into (1) and (2), *d*_*ij*_ and *d*_*av*_^*i*^ become dimension-less quantities. The ECN_*i*_ values are dimension-less regardless of whether or not Eq. (3) is used.


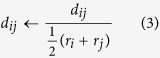


The average ECNs and variances of atomic charges were calculated using Eqs. (4) and (5), respectively. A large variance of atomic charge means the cluster is strongly polarized in the geometry.


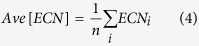



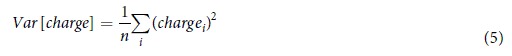


To understand the differences in the adsorption interactions between ORR intermediates O/OH and each sub-nanosized cluster, we developed an AIMC simulation code by ourselves using Python with the NumPy module^44^. The detailed procedure of the AIMC simulation is as follows. First, we selected a set of sub-nanosized cluster geometries from pre-performed AIMD-SA simulations by random sampling with energy weight (Boltzmann factor). Second, an adsorbed molecule of O/OH was placed randomly near the surface of the cluster selected in the first step. In this step, the shortest distances between the adsorbed molecule and cluster were defined to be those in the 1.3–3.0 Å range. The geometries that did not satisfy the requirement were not adopted from the AIMC simulations. Then, using the accepted geometries, geometry optimizations were performed using the quasi-Newton-Raphson method with convergence criteria of 10^−6^ a.u. For all sub-nanosized clusters considered in this study, we obtained 10^5^ adsorption geometries with O/OH. Finally, the interaction energies between the sub-nanosized clusters and adsorbates were evaluated. In the geometry optimizations, all atoms were fully relaxed to allow geometry changes through adsorption interactions. In the final complex geometries, the interaction energies were calculated using Eq. (6).


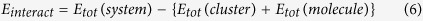


Further details of the AIMC procedure are described in the Supporting Information.

## Additional Information

**How to cite this article:** Miyazaki, K. and Mori, H. Origin of high oxygen reduction reaction activity of Pt_12_ and strategy to obtain better catalyst using sub-nanosized Pt-alloy clusters. *Sci. Rep.* **7**, 45381; doi: 10.1038/srep45381 (2017).

**Publisher's note:** Springer Nature remains neutral with regard to jurisdictional claims in published maps and institutional affiliations.

## Supplementary Material

Supplementary Information

## Figures and Tables

**Table 1:** Energy-weighted averages and standard deviations of adsorption energies in cluster-O/OH (cluster = Pt_12_, Pt_13_, Al_4_Pt_8_, Ga_4_Pt_8_, Ge_4_Pt_8_, and Sn_4_Pt_8_) in units of eV.

	Pt_12_	Pt_13_	Al_4_Pt_8_	Ga_4_Pt_8_	Ge_4_Pt_8_	Sn_4_Pt_8_
O	−3.00 ± 0.17	−2.80 ± 0.05	−3.53 ± 0.17	−2.33 ± 0.06	−3.18 ± 0.22	−2.28 ± 0.12
OH	−4.17 ± 0.27	−4.11 ± 0.03	−4.58 ± 0.13	−3.21 ± 0.10	−3.78 ± 0.30	−3.06 ± 0.19

**Table 2:** Vibrational wavenumbers/periods of MPt (M = Pt, Al, Ga, Ge, and Sn) and time steps applied in AIMD-SA simulations of Pt_12_, Pt_13_, and M_4_Pt_8_ (M = Al, Ga, Ge, and Sn).

	Pt_2_	AlPt	GaPt	GePt	SnPt
Vibrational wavenumber [cm^−1^]	218.4^a^	379.6	234.5	325.71	254.41
Period [fs]	152.6	87.9	142.1	102.3	131.0
Timesteps in AIMD-SA [fs]	10	5	10	10	10

^a^Reference 45.
