# Supplementary material for: Origin of high oxygen reduction reaction activity of Pt12 and strategy to obtain better catalyst using sub-nanosized Pt-alloy clusters
Source: Sci Rep. 2017 Mar 28;7:45381. doi: 10.1038/srep45381 (PMC5368974; doi:10.1038/srep45381)
Supplement: Supplementary Information [file srep45381-s1.pdf]

Supplementary Information

Origin of high oxygen reduction reaction activity of Pt_{12} and strategy to obtain better catalyst using sub-nanosized Pt-alloy clusters

Kasumi Miyazaki[1] and Hirotoshi Mori[2]*

[1]Department of Chemistry and Biochemistry, Graduate School of Humanities and Sciences, Ochanomizu University, 2-1-1 Otsuka, Bunkyo-ku, Tokyo 112-8610, Japan

[2]Faculty of Core Research Natural Science Division, Ochanomizu University, 2-1-1 Otsuka, Bunkyo-ku, Tokyo 112-8610, Japan

*Corresponding Author E-mail: mori.hirotoshi@ocha.ac.jp

1. Atomic natural charge in global minimum geometries of Pt_{12}, Pt_{13}, Al_4Pt_8, Ga_4Pt_8, Ge_4Pt_8 and Sn_4Pt_8.

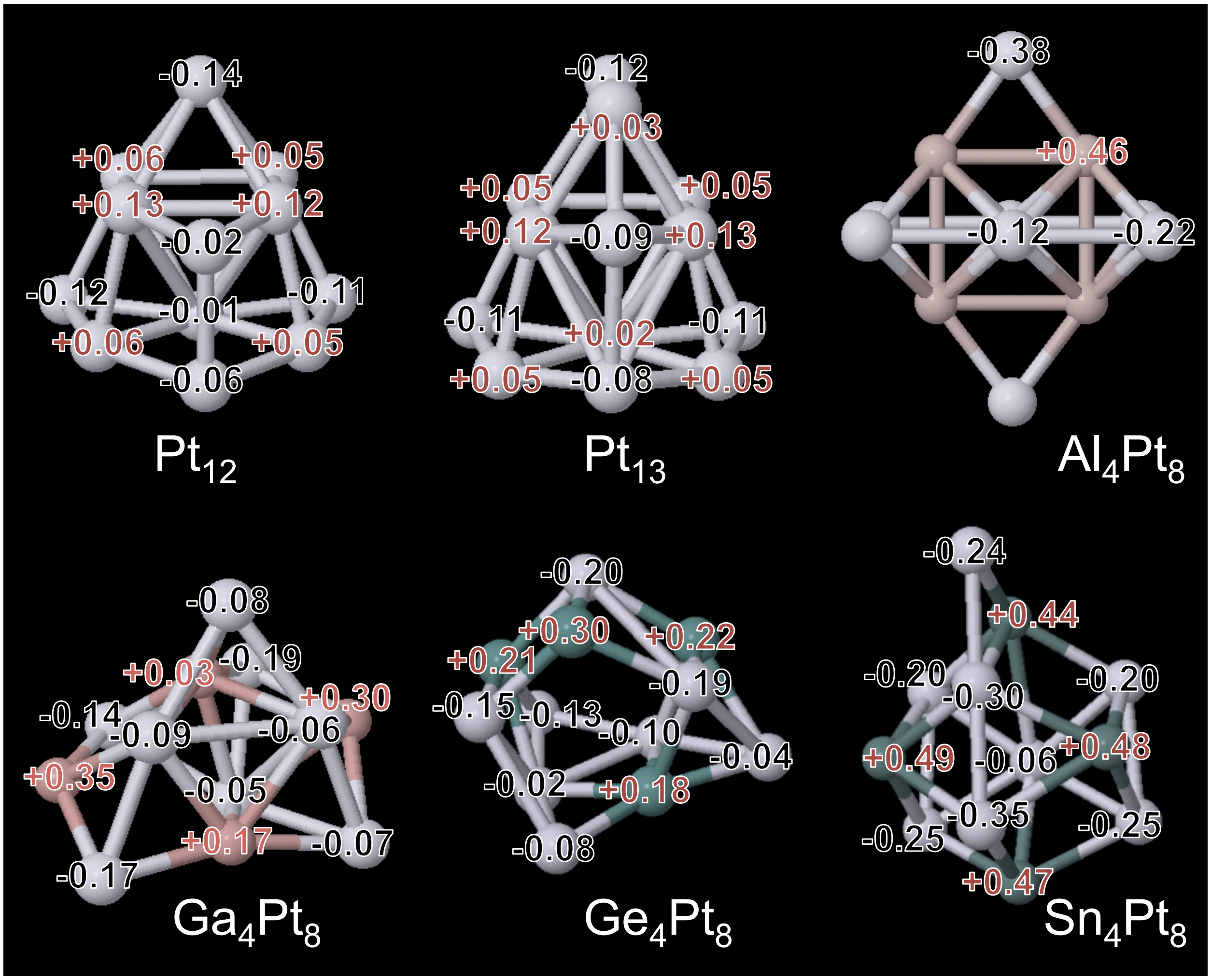

Figure S1. Atomic charge in global minimum geometries.

2. Density of states (DOS).

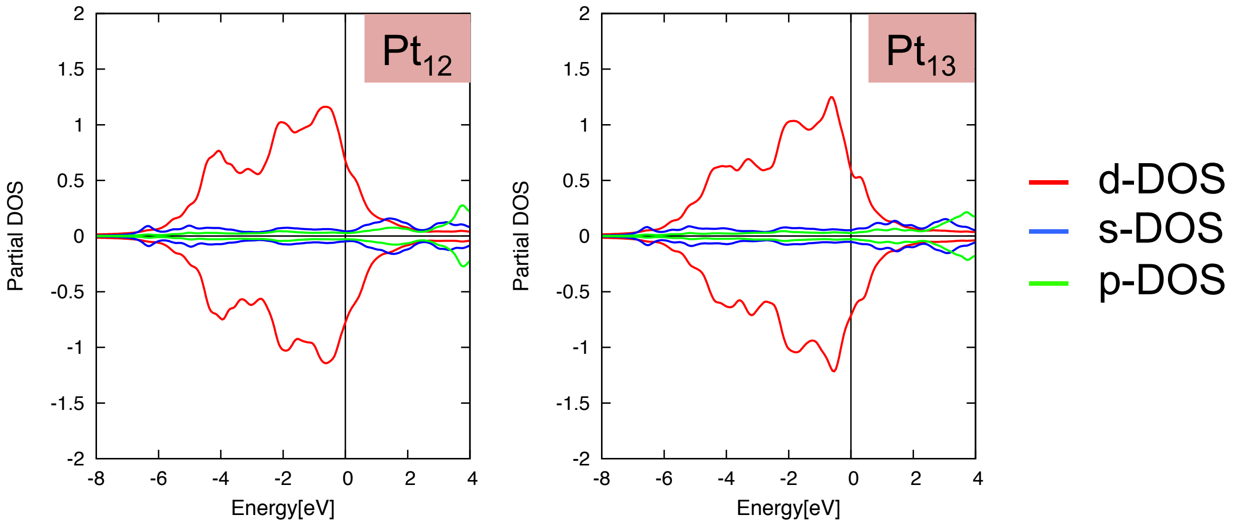

Figure S2. Partial DOS in global minimum geometries of Pt_{12} and Pt_{13}.

3. Adsorption energy of O/OH for alloy clusters.

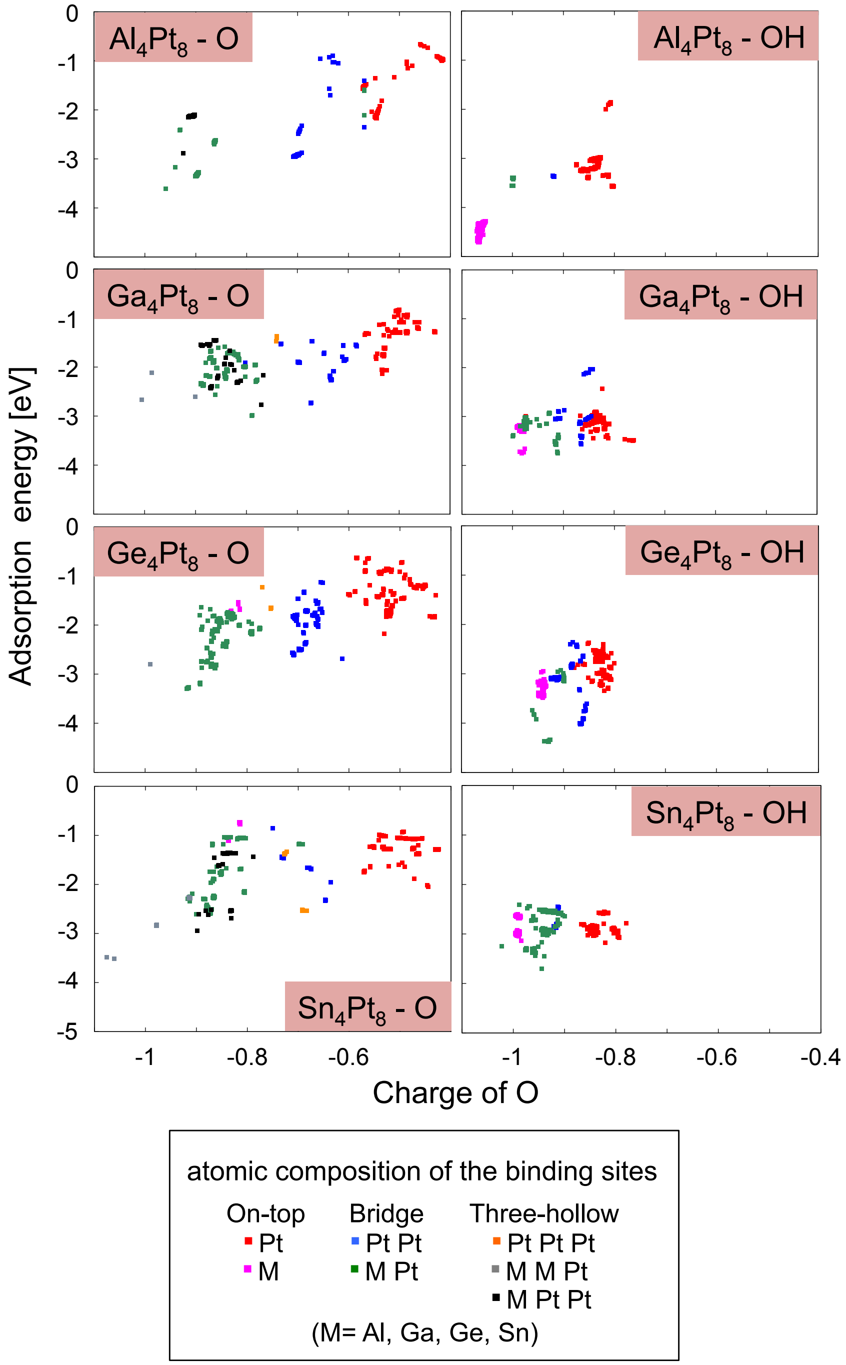

Figure S3. Correlation map adsorption energy with natural charge on an oxygen atom in M_4Pt_8…O/OH (M = Al, Ga, Ge, Sn). Data for different adsorption sites were plotted with different colors.

4. Effective coordination number (ECN) in global minimum geometries of Pt_{12}, Pt_{13}, Al_4Pt_8, Ga_4Pt_8, Ge_4Pt_8 and Sn_4Pt_8.

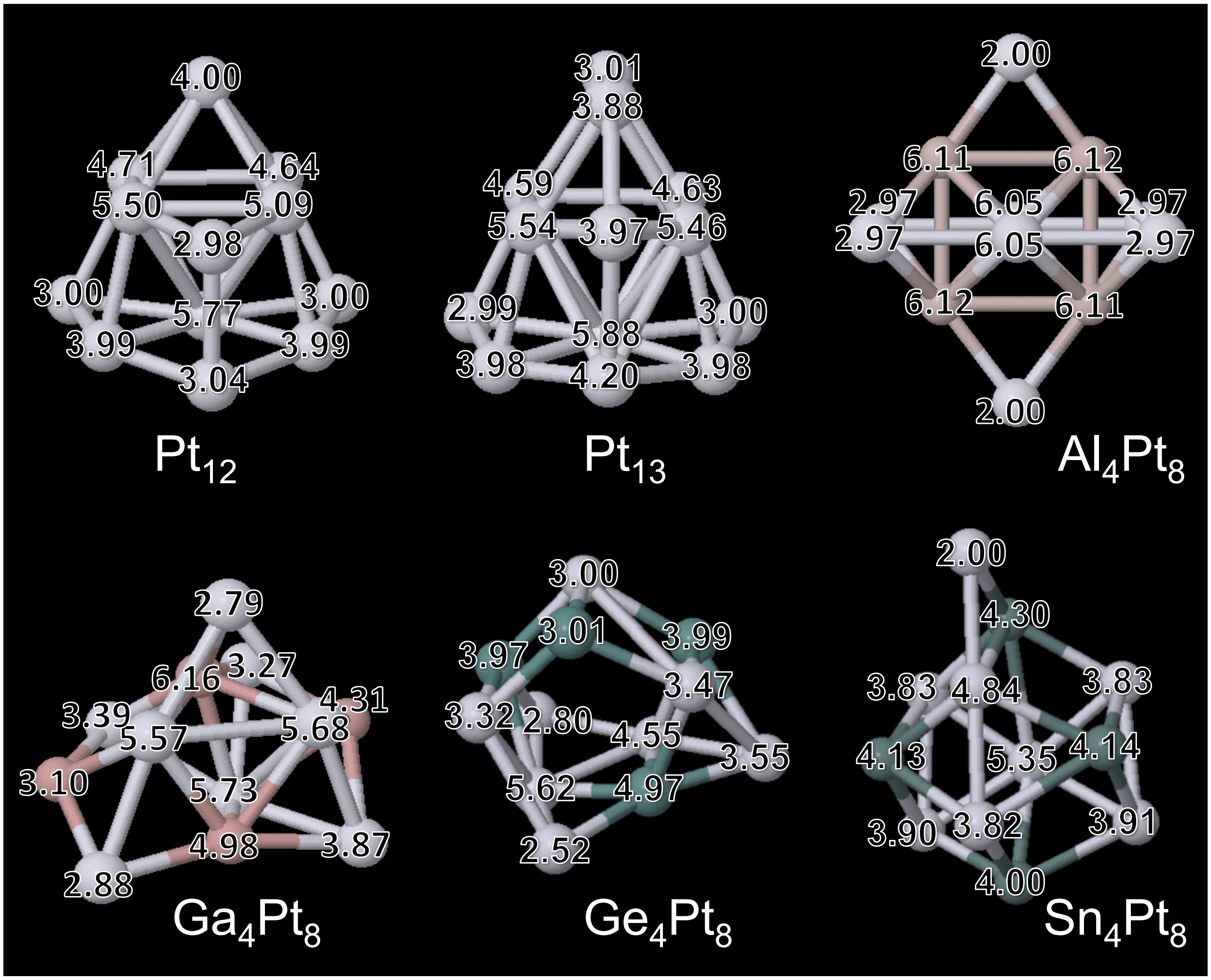

Figure S4. Effective coordination number (ECN) in global minimum geometries.

5. Details of *ab initio* Monte Carlo (AIMC).

AIMC scheme is as follows.

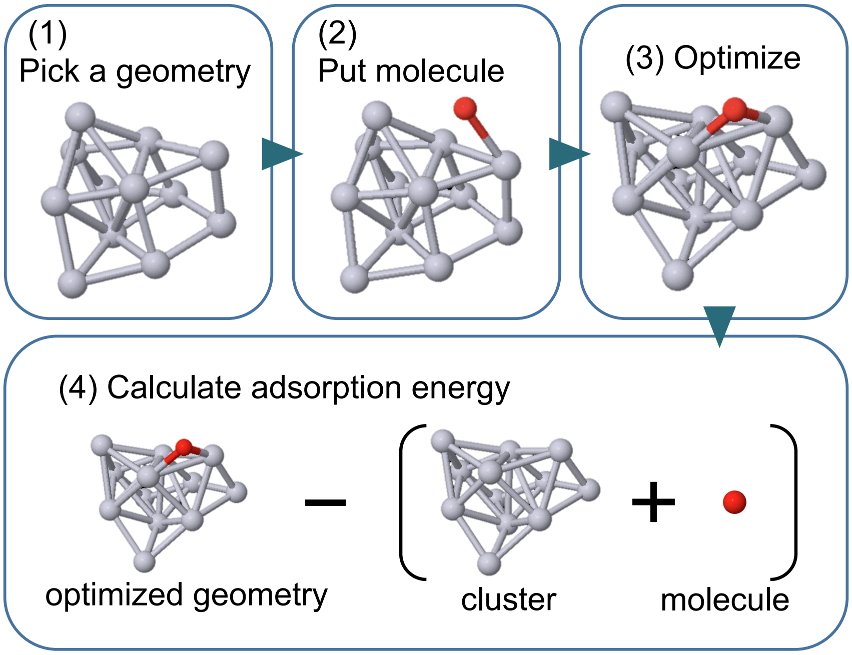

Figure S5. Procedure of AIMC.

(1) A cluster geometry from the AIMD-SA simulations was picked up using energy-weighted random sampling. The weight w_i for geometry i was defined by Boltzmann factor (see Eq. (S1)). Here, ΔE_i, k and T are relative energy of geometry i, Boltzmann constant, and the absolute temperature, respectively. To obtain many samples from wide range of energy, we set T = 1000K.

$$w_i = \exp\left(\frac{-\Delta E_i}{kT}\right) \tag{S1}$$

(2) The center of mass of the cluster (***G***) was defined as an origin of the Cartesian coordinate. Then, an adsorbate is put randomly on the cluster surface, which is in the range of 4-7Å from ***G***.

As shown in **Figure S6**, in the case that the adsorbate is an O atom, the Cartesian coordinates of the O atom was defined by Eq. (S2), where $\mathrm{rand}(a, b)$ and $\boldsymbol{A}(x, y, z)$ are random floating-point number n ($a \leq n \leq b$) and the Cartesian coordinate of the adsorbate O, respectively.

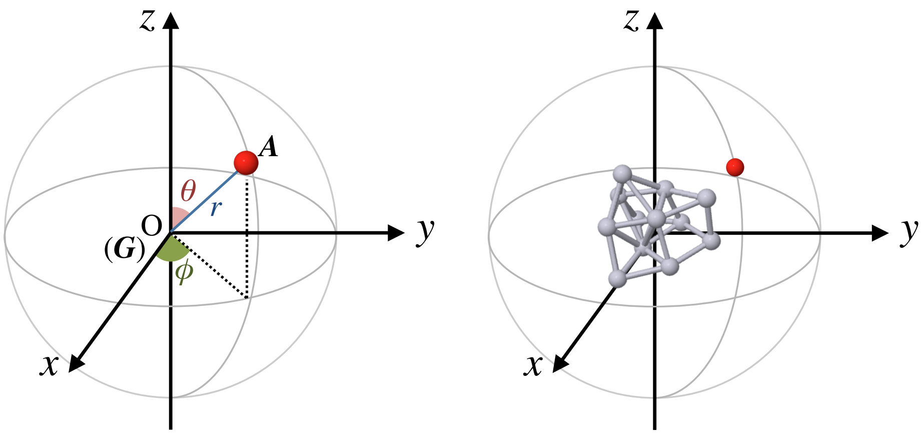

Figure S6. Generation of random configuration of O.

$$r = \sqrt[3]{\mathrm{rand}(4^3, 7^3)} \qquad \cos\theta = \mathrm{rand}(-1, 1) \qquad \phi = \mathrm{rand}(0,\, 2\pi) \qquad \boldsymbol{A} = \begin{bmatrix} r\sin\theta\cos\phi \\ r\sin\theta\sin\phi \\ r\cos\theta \end{bmatrix} \tag{S2}$$

In the case that adsorbate is diatomic OH molecule, we followed a similar procedure to the above case. In this case, additionally, we have to take care about OH orientation to the cluster surface. The final OH coordinates were defined by Eq. (S3). The definitions of the variables in the Eq. (S3) are shown in **Figure S7**.

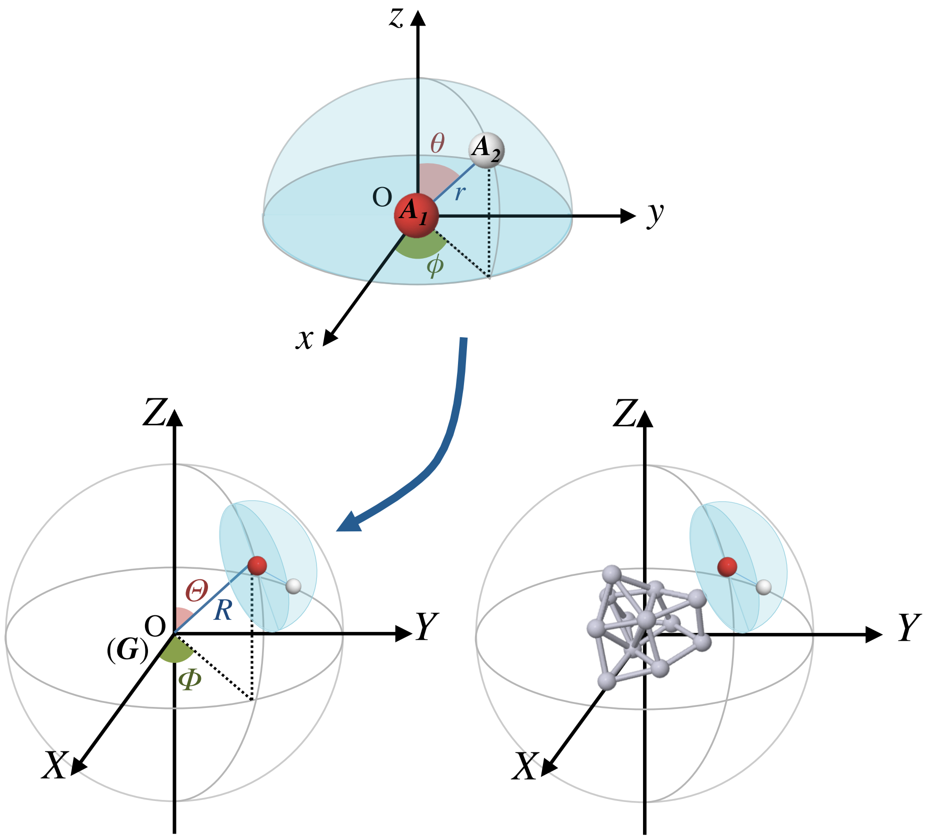

Figure S7. Generation of random configuration in the case of dimer.

$$r = 0.97\text{Å (optimum bond length of OH)}$$

$$\cos\theta = \mathrm{rand}(0, 1)$$

$$\phi = \mathrm{rand}(0, 2\pi)$$

$$R = \sqrt[3]{\mathrm{rand}(4^3, 7^3)}$$

$$\cos\Theta = \mathrm{rand}(-1, 1) \tag{S3}$$

$$\Phi = \mathrm{rand}(0, 2\pi)$$

$$\boldsymbol{A}_1 = \begin{bmatrix} 1 & 0 & 0 \\ 0 & \cos\Theta & \sin\Theta \\ 0 & -\sin\Theta & \cos\Theta \end{bmatrix} \begin{bmatrix} \cos\Phi & \sin\Phi & 0 \\ -\sin\Phi & \cos\Phi & 0 \\ 0 & 0 & 1 \end{bmatrix} \begin{bmatrix} 0 \\ 0 \\ R \end{bmatrix}$$

$$\boldsymbol{A}_2 = \begin{bmatrix} 1 & 0 & 0 \\ 0 & \cos\Theta & \sin\Theta \\ 0 & -\sin\Theta & \cos\Theta \end{bmatrix} \begin{bmatrix} \cos\Phi & \sin\Phi & 0 \\ -\sin\Phi & \cos\Phi & 0 \\ 0 & 0 & 1 \end{bmatrix} \begin{bmatrix} r\sin\theta\cos\phi \\ r\sin\theta\sin\phi \\ r\cos\theta + R \end{bmatrix}$$

(3) The shortest Pt-O distance in the system was checked whether it was in range of 1.3-3.0Å. Configurations that satisfy the condition were optimized by quasi Newton-Raphson method with a criterion 10^{-6} a.u. While the geometry optimizations, all atoms were relaxed.

(4) Adsorption energy was calculated for each optimized system by Eq. (S4).

$$E_{interact} = E_{tot}(complex) - \{E_{tot}(cluster) + E_{tot}(molecule)\} \tag{S4}$$

6. Atomic configurations of global and relatively stable local minimum geometries of Pt_{12}, Pt_{13}, Al_4Pt_8, Ga_4Pt_8, Ge_4Pt_8 and Sn_4Pt_8 in units of Å.

The labels, “a, b, ... , m” correspond to that of Figure 2.

```
12
label_a
Pt   3.3356669  -28.2112444   20.1418341
Pt   3.6836091  -24.9178981   16.2949426
Pt   1.0838054  -25.9812516   18.6813477
Pt   1.8574939  -23.7338485   17.7344875
Pt   3.2586638  -27.0498343   17.7950631
Pt   0.8899686  -27.6895339   20.6840308
Pt   4.2367078  -24.9443997   18.8802409
Pt   0.0161094  -23.6825699   19.7206753
Pt   2.5794292  -22.9516049   20.2641681
Pt   1.8018011  -25.2428674   21.0770136
Pt   4.2613262  -26.0079714   21.2149033
Pt   5.0405548  -23.4979079   20.9375181
```

```
12
label_b
Pt   -9.6281587  -20.4826957  -23.1766063
Pt   -8.2765312  -15.5749890  -21.8200554
Pt  -10.8504785  -18.2433881  -19.5065691
Pt  -11.7411235  -19.2521926  -21.8057096
Pt   -9.3932749  -20.2867870  -20.3924341
Pt  -10.4235410  -17.0771102  -22.0274169
Pt  -11.7175144  -19.5612211  -24.4093948
Pt   -9.6734254  -17.9462862  -24.3360704
Pt   -8.3535385  -17.9887049  -20.7771907
Pt   -7.4484754  -16.6616348  -23.9795704
Pt   -7.5625824  -21.4017514  -21.9039960
Pt   -7.2726933  -19.0063556  -22.8856833
```

```
12
label_c
Pt  -2.9761179   0.0000000  -1.2845518
Pt  -0.8218913  -1.4235574  -1.2806050
Pt   1.4880590  -2.5773937  -1.2845518
Pt   1.6437825   0.0000000  -1.2806050
Pt  -0.8218913   1.4235574  -1.2806050
Pt   1.4880590   2.5773937  -1.2845518
Pt  -2.9761179   0.0000000   1.2845518
Pt  -0.8218913  -1.4235574   1.2806050
Pt   1.6437825   0.0000000   1.2806050
Pt   1.4880590  -2.5773937   1.2845518
Pt  -0.8218913   1.4235574   1.2806050
Pt   1.4880590   2.5773937   1.2845518
```

```
12
label_d
Pt   9.3068936   20.3944484   23.1635108
Pt   7.2743246   23.7343331   20.0348819
Pt   9.0202455   21.8998030   20.4569214
Pt   6.8393384   20.4783524   22.3714330
Pt   4.7000397   21.8059488   21.6795507
Pt   5.6407820   21.8556058   19.2211910
Pt   9.4163166   23.0148360   22.7756163
Pt   8.2696692   25.2612299   21.9599753
Pt   7.3853683   20.0096143   19.8719257
Pt   5.4790890   21.6973824   24.2614840
Pt   6.8511034   23.0613137   22.4752580
Pt   9.7862276   19.4091893   20.8239953
```

```
12
label_e
Pt  14.2592193   11.5551216  -24.2760560
Pt  12.7976349    9.2903053  -25.8568006
Pt  14.7343909   10.7730226  -26.6913038
Pt  11.9528669   12.3371812  -25.0955021
Pt  12.2686068   11.3178513  -27.4528620
Pt  10.7740257    8.0297231  -24.8426896
Pt  12.3911393   12.2521044  -22.4263520
Pt  12.4156841    7.1219741  -23.0885505
Pt  14.5209315    8.0657488  -24.3283533
Pt  13.1922738    9.6733059  -22.8299336
Pt  16.3086575    9.9034505  -24.7721433
Pt  10.8884732   10.3790749  -23.7354579
```

```
12
label_f
Pt   2.2105425   3.1786454   3.7670234
Pt   4.4216200   2.3388710   1.0965166
Pt   2.1882914   5.8165650   4.0394458
Pt   5.5507225   5.9117789   1.7086036
Pt   1.5670877   3.0128694   1.1436896
Pt   6.0299011   4.0426935  -0.0948906
Pt   2.5967636   0.9745308   2.3760092
Pt   4.7105166   6.3075070   4.2275028
Pt   3.5071534   4.2769487  -0.6778921
Pt   1.0469070   5.1305249  -0.2587633
Pt   2.9319063   5.3086759   1.5945370
Pt   4.5992549   3.9441068   3.1014062
```

12

label_g

Pt	-26.0909417	2.2756026	16.7522971
Pt	-28.8271294	1.9772353	18.6280870
Pt	-26.7429618	3.5425029	18.8860422
Pt	-28.2042293	0.8184300	16.2706358
Pt	-23.1175435	0.5634171	16.1751994
Pt	-26.8479025	0.2929501	18.5041742
Pt	-25.4612766	-0.0949574	20.6686298
Pt	-24.1965522	-0.3608423	18.3737076
Pt	-24.3690814	4.6428256	19.0493559
Pt	-23.6447960	2.9449424	17.1638888
Pt	-24.5299157	2.0014175	19.3880144
Pt	-25.6315564	-0.1185940	15.9000979

12

label_h

Pt	9.9968610	16.7426937	-0.7871627
Pt	9.2113332	14.6513367	2.0628916
Pt	9.2456077	14.7643094	-2.5410116
Pt	6.7513916	13.7811893	1.8164321
Pt	11.3176504	15.9580555	1.3372023
Pt	9.8971681	12.3067993	-1.8802100
Pt	8.3978178	12.5173979	0.2172299
Pt	7.9347317	15.0617251	-0.1870173
Pt	11.5139266	14.2093842	3.3059315
Pt	10.0939782	10.4853928	0.0192570
Pt	10.9100833	12.4080015	1.5483529
Pt	10.9283015	14.1213669	-0.4021312

12

label_i

Pt	-7.1304464	24.5391936	-18.9558767
Pt	-3.3517699	26.7225860	-20.5253078
Pt	-4.0778403	24.5431986	-21.7660680
Pt	-5.2267857	22.4967445	-20.1240887
Pt	-5.4594763	23.0681465	-17.5817645
Pt	-8.4613058	25.9177169	-20.7081491
Pt	-7.8358594	22.3684738	-20.2276603
Pt	-5.9589347	26.5776444	-20.5921550
Pt	-7.0274708	24.1212583	-21.9746627
Pt	-5.1854451	22.4021980	-22.7169191
Pt	-5.7197981	26.0528558	-23.1469037
Pt	-4.3301013	24.9172502	-18.970032

13

label_j

Pt	26.5910856	-8.9309319	20.1197244
Pt	29.4703662	-9.9127348	22.0175689
Pt	30.6891796	-11.2239787	18.1964313
Pt	27.2057482	-8.6954259	22.6349241
Pt	28.3781145	-10.1224934	17.0725438
Pt	25.9176922	-9.4906881	17.6543483
Pt	28.1882837	-12.0487315	18.7768034
Pt	25.7041471	-13.2553351	21.2393422
Pt	28.9940511	-9.5993440	19.5226653
Pt	25.7518135	-11.4823393	19.3405158
Pt	27.2549101	-11.2057461	21.8246518
Pt	30.5501855	-11.9658400	20.7623362
Pt	28.3316230	-13.3773408	20.9616395

13

label_k

Pt	-10.3571769	10.4838317	8.1925276
Pt	-8.1924065	11.7529621	12.5807757
Pt	-11.1501306	11.2773859	10.5875913
Pt	-8.6572453	10.7508201	10.2089898
Pt	-6.1950738	11.4115206	9.6293807
Pt	-10.6470964	12.6251612	12.8017338
Pt	-7.3336994	13.4396664	10.7796471
Pt	-8.9861488	12.6713996	8.2043402
Pt	-7.3364586	14.7021486	8.5259985
Pt	-8.5892992	15.7797522	10.5763943
Pt	-6.5115503	9.9542822	11.7529345
Pt	-10.0044889	13.6247844	10.4438337
Pt	-6.5130484	12.5460806	7.2984957

13

label_l

Pt	-15.0333636	19.4772664	-1.1860497
Pt	-16.3422169	22.3038616	-3.7219370
Pt	-18.1953658	20.1543535	-3.2595171
Pt	-18.8641433	22.4505927	-4.3052440
Pt	-15.6778823	22.2590180	-0.9605649
Pt	-14.5713075	21.3503489	-5.3578877
Pt	-13.3529767	21.2919691	-0.3377512
Pt	-18.2171471	22.6814496	-1.7998759
Pt	-13.8772126	21.5741143	-2.8652631
Pt	-17.0505459	18.0418829	-2.1404231
Pt	-17.6483100	22.3939131	0.7180646
Pt	-15.6759091	19.5073272	-3.8714460
Pt	-17.5572540	20.1208282	-0.5715388

13
label_m
Pt 7.3636013 14.0803568 -8.6629553
Pt 7.0280691 14.8437100 -11.1361861
Pt 8.9020874 15.8697622 -12.9926824
Pt 9.2810815 15.8862896 -8.7991090
Pt 4.6423130 13.8224274 -10.9670445
Pt 9.3726477 12.4293706 -9.3344046
Pt 8.1321088 17.1584862 -10.7977301
Pt 9.6777002 14.4413677 -10.8868320
Pt 5.4757903 12.3434071 -8.9920064
Pt 9.5539296 13.8665793 -7.1493266
Pt 6.7385424 12.2511950 -11.3011240
Pt 8.7610517 13.1477353 -12.9146880
Pt 8.9925383 10.9823101 -11.451729

12
Al4Pt8
Pt 20.4010981 -0.1130227 -43.5826223
Pt 21.8092644 3.8836032 -43.1609438
Pt 16.9151933 2.2726597 -44.1257703
Al 18.8021804 1.1481812 -45.2352687
Pt 19.3338497 3.1638282 -43.6434095
Pt 17.9256835 -0.8327976 -44.0650880
Al 18.2991238 1.0359957 -42.5102448
Pt 20.4643842 1.6514854 -46.8406317
Al 20.9327674 1.9026244 -41.9907631
Pt 19.2705636 1.3993202 -40.3854001
Pt 22.8197545 0.7781459 -43.1002615
Al 21.4358240 2.0148099 -44.7157870

12
Ga4Pt8
Pt 2.9485401 -27.2841832 19.3318784
Pt 4.2835322 -24.8223772 16.4002221
Ga 0.8896381 -25.8572425 19.4557607
Pt 1.9864279 -24.3743017 17.5856686
Ga 2.8896989 -26.7377254 16.9716498
Pt 0.5789080 -27.9524032 20.6435394
Ga 4.2596440 -25.1977212 18.8761934
Pt 0.1491827 -23.5814380 19.3526174
Pt 2.6419757 -23.6717412 20.2568002
Pt 2.0992767 -26.0422766 21.6953274
Ga 4.0430859 -24.7273732 22.3310424
Pt 5.2752170 -23.6620632 20.5254594

12
Ge4Pt8
Ge 16.3409698 -17.1190435 7.5058065
Ge 16.3938001 -13.6240730 6.9383986
Ge 18.3220976 -16.3388162 5.0568721
Ge 18.8265512 -14.6801168 8.6617973
Pt 16.0264113 -15.6550300 5.5468635
Pt 19.6188793 -14.3959452 4.4368999
Pt 20.1256176 -15.5900274 6.7277119
Pt 16.4238024 -15.1514755 8.9225497
Pt 19.9485594 -16.7274655 9.1272621
Pt 17.6555431 -12.5580336 9.0036818
Pt 18.7416457 -13.1263218 6.6132425
Pt 18.5470597 -17.8756756 7.0073410

12
Sn4Pt8
Sn -4.8170813 -10.4751726 11.1204529
Sn -4.8215457 -13.2286375 6.5598718
Sn -5.8807850 -13.9842934 10.0019826
Sn -6.4860001 -10.1407638 7.6827459
Pt -7.2053759 -12.9066479 7.7128311
Pt -3.5327188 -11.8045029 8.5768127
Pt -4.1294831 -9.2918244 8.8313136
Pt -3.5730467 -12.8288444 10.9633236
Pt -7.1468037 -10.1910398 12.1711098
Pt -7.1307210 -11.5288114 9.9729163
Pt -4.1669329 -10.6084111 6.2992651
Pt -3.5561554 -14.4541056 8.6235225
